# Perceived cardiovascular disease risk and tailored communication strategies among rural and urban community dwellers in Rwanda: a qualitative study

**DOI:** 10.1186/s12889-022-13330-6

**Published:** 2022-05-09

**Authors:** Jean Berchmans Niyibizi, Kufre Joseph Okop, Jean Pierre Nganabashaka, Ghislaine Umwali, Stephen Rulisa, Seleman Ntawuyirushintege, David Tumusiime, Alypio Nyandwi, Evariste Ntaganda, Peter Delobelle, Naomi Levitt, Charlotte M. Bavuma

**Affiliations:** 1 College of Medicine and Health Sciences (CMHS), University of Rwanda, Kicukiro Campus, KK19 Av 101, P.O. Box 4285, Kigali, Rwanda; 2 Chronic Diseases Initiative for Africa (CDIA), Department of Medicine, University of Cape Town, Cape Town, South Africa; 3 Kigali University Teaching Hospital, Kigali, Rwanda; 4 Ministry of Health, Kigali, Rwanda; 5 Rwanda Biomedical Centre, Kigali, Rwanda; 6 Department of Public Health, Vrije Universiteit Brussel, Brussels, Belgium

**Keywords:** Cardiovascular disease, Perceived risk, Communication strategies, Rwanda

## Abstract

**Background:**

In Rwanda, cardiovascular diseases (CVDs) are the third leading cause of death, and hence constitute an important public health issue. Worldwide, most CVDs are due to lifestyle and preventable risk factors. Prevention interventions are based on risk factors for CVD risk, yet the outcome of such interventions might be limited by the lack of awareness or misconception of CVD risk. This study aimed to explore how rural and urban population groups in Rwanda perceive CVD risk and tailor communication strategies for estimated total cardiovascular risk.

**Methods:**

An exploratory qualitative study design was applied using focus group discussions to collect data from rural and urban community dwellers. In total, 65 community members took part in this study. Thematic analysis with Atlas ti 7.5.18 was used and the main findings for each theme were reported as a narrative summary.

**Results:**

Participants thought that CVD risk is due to either financial stress, psychosocial stress, substance abuse, noise pollution, unhealthy diets, diabetes or overworking. Participants did not understand CVD risk presented in a quantitative format, but preferred qualitative formats or colours to represent low, moderate and high CVD risk through in-person communication. Participants preferred to be screened for CVD risk by community health workers using mobile health technology.

**Conclusion:**

Rural and urban community members in Rwanda are aware of what could potentially put them at CVD risk in their respective local communities. Community health workers are preferred by local communities for CVD risk screening. Quantitative formats to present the total CVD risk appear inappropriate to the Rwandan population and qualitative formats are therefore advisable. Thus, operational research on the use of qualitative formats to communicate CVD risk is recommended to improve decision-making on CVD risk communication in the context of Rwanda.

**Supplementary Information:**

The online version contains supplementary material available at 10.1186/s12889-022-13330-6.

## Background

Non-communicable diseases (NCDs) is one of the major public health concerns around the world, with a disproportionately NCD burden on Low- and middle- income countries (LMICs). In 2017, the analysis of ‘World Health Organization (WHO) Global Health Estimates’ mortality data revealed that people living in LMICs have a 1.5 times higher risk of premature NCD death than people living in high-income countries (HICs) [1].

This relative risk of premature NCD death is an average of four main NCD subtypes (cardiovascular diseases, all cancers, diabetes and respiratory chronic diseases), however, it is higher for some subtypes such as cardiovascular diseases (CVD) as revealed by recent studies. Globally, in 2017, 17.8 million people died of CVD [2]. Low- and middle- income countries (LMICs) were more affected as the rate of CVD death was 2.5 times higher than in high-income countries (HICs) [2]. Before the 1990’s, HICs accounted for the highest age-standardized CVD mortality rate globally, but this has declined in the last decades due to the impact of preventive interventions [2].In LMICs, there is therefore a need to develop evidence-based and robust prevention strategies in order to stop or reverse the emerging CVD burden.

Rwanda, as one of LMICs located in East Africa, has been likewise affected by the current emerging CVD burden. For instance, in 2013, the Word Health Organization (WHO)-STEPS survey on non-communicable diseases (NCDs) risk factors in Rwanda found that 19.1% of men and 7.1% of women were smokers, 30.0% of men and 17.0% of women were heavy drinkers (of alcohol) at the time of interview, and 99.1% of the population reported low fruit and vegetable intake [3]. The Rwanda Demographic and Health Survey (RDHS) 2014-15 reported that 17.1 % and 3.7% of women aged 15-49 in Rwanda are overweight and obese, respectively [4]. The same survey reported that 5.8% and 0.4% of men in age range of 15-49 or 15-59 in Rwanda are overweight and obese, respectively [4]. Furthermore, CVD was the third leading cause of death, responsible for approximately 7.5% of total annual deaths in 2012 [3]. Recently Rwanda vital statistics report of 2020 revealed that NCDs, which include CVD, represented 34.7% of usable death causes [5].

The strategy of identifying and managing individuals at high risk of CVD is paramount for CVD prevention and control, in addition to other multi-sectoral population-based interventions targeting CVD risk factors in the entire population.[6] The prevention of CVD requires population awareness of the risk for developing CVD and risk reduction strategies [7]. Awareness and understanding of personal risk is an important step for adopting healthy behaviors aimed at preventing ill-health, including CVD events [7, 8].This is in line with the constructs of the health belief model (HBM) as described by Jones et al. [9], which state that an accurate evaluation of health risk is one of the critical factors of perceived personal vulnerability to such risk, which in turn informs health-seeking behavior. In the HBM model, self-evaluation of CVD risk is considered as a function of an individual’s perception of vulnerability, understanding of the threat, the effect of societal motivations / demotivations and the perceived benefits of taking action to avert such risk [9].

The way in which information is conveyed plays an important role for understanding the level of CVD risk, which in turn determines health seeking behavior for risk prevention [9, 10]. Graphic formats or bar charts and thermometer formats (with percentages) have, for example, been evaluated in previous qualitative studies conducted in Australia and their results are inconclusive to some degree [4, 11]. This indicates the need for more contextualized research into CVD risk presentation formats, as results may differ from one setting to another. A specific need for research in CVD risk communication, including development of standardized tools that can be used by health workers, was indeed previously highlighted [7].

To the best of our knowledge no other study has been conducted in Rwanda in regard to CVD risk communication. This study was undertaken to explore the understanding of, and perception towards, CVD risk and CVD risk communication methods among rural and urban community dwellers to inform evidence-based policies and practices in Rwanda.

## Methods

### Study design

The study used an exploratory qualitative design to explore community members’ understanding of self-perception and tailored communication strategies towards CVD risk among rural and urban dwellers. In this study, the term ‘community’ was operationalized as the catchment area of a health center (HC) providing NCDs (including CVD) services.

### Setting, study population and sampling strategy

One HC in Burera district, Northern Province and one HC in Gasabo district, City of Kigali were selected as rural and urban study sites, respectively. The study population included people with limited education (completed secondary education and below) and aged 18-65 years. This study targeted community members in the age range of 18-65 because it is quite similar age group (15-64) considered in the WHO-STEPS survey on non-communicable diseases (NCDs) risk factors in Rwanda, conducted in 2013 [3]. To ensure that all study participants can give their consent, community members under the age of 18 were excluded.

Data were collected through focus group discussions (FGDs) with community members recruited from the local community. FGDs were preferred over interviews because the main objective of the study was to explore what local community members perceive that could potentially put them at CVD risk. This objective can be adequately achieved in case community members’ opinion or perception feed off opinions / perceptions from other fellow community members. In other words, FGDs are thought to be the best way to exchange viewpoints among local community members and thus this helps to maximally explore opinions and perceptions from study participants. In addition to this, the questions set to achieve the objective were not sensitive at the extent to which FGD participants would not express themselves in presence of other fellow community members. Thus, FGD was rational over interviews. In total, eight FGDs were conducted for the entire study, four in each study site (rural / urban). For the sake of homogeneity among participants, FGDs were divided by gender (men and women) and age group (young: 18-24 years old and adult: 25-65 years old) to enhance participants’ comfort and relevance to their life stage. In total, four FGDs were conducted in each site (rural / urban), including two FGDs with youth (one with men and one with women), and two FGDs with adults (one with men and one with women).

A quota sampling strategy was used to make sure that four sub-populations per sex and age groups, as mentioned above, are represented during this study. Community members fulfilling eligibility criteria were recruited within the catchment area of the selected HCs. They were selected using the existing forums for social cohesion at village level, including young people forums and parents’ evening forums which are held monthly in each village. In doing so, one cell (the basic political-administrative unit composed by villages in Rwanda) was randomly selected in the catchment area of each selected HC, followed by the selection of two villages within each cell. The first village served to recruit participants of FGDs with youth and the second village served to recruit participants of FGDs with adults.

During the recruitment phase, two members of research team (JBN and JPN or GU) attended parents evening forums and young people forums in the selected villages to recruit potential study participants. In doing so, they explained about the study to the community members who attended the targeted forum (parents evening forum or young people forum at village level), then requested first of all men who were interested in participating in the study to put their hands up. Researchers attentively observed how community members were putting up their hands until the 20^th^ person who put his /her hand up. One of the eligibility criteria for participating in FGDs was an adequate level of literacy and numeracy. The first 20 community members who voluntarily manifested interest in participating in the study were tested against these criteria. Research team agreed each other to test the numeracy and literacy of community members who voluntarily accepted to be part of the study. Each potential study participant was given a piece of paper with three fractions: “i) 5%, ii) 10% and iii) and 20%”, he / she was requested to read them loudly in local language (Kinyarwanda) for numeracy test. For literacy test, each potential participant was given a piece of paper on which the following sentence was written: “The likelihood of you having a particular disease is five or more times than that of your friend or neighbor of the same age” that was translated into local language (Kinyarwanda). The participant was requested to read aloud the sentence. Both numeracy and literacy tests were conducted by one researcher who judged the level of numeracy and literacy for each tested community member and put potential study participants on the order depending on how their level of numeracy and literacy skills was judged. The first 12 community members who were judged to have more adequate numeracy and literacy skills than others were retained and immediately had a short meeting with researchers to agree each other on the date and venue of FGD. Local community members with poor literacy skills were excluded because data collection was set in the way each FGD participant should be given a piece of paper and requested to interpret it according to what they read.

### Data collection

Data were collected using a FGD guide that was developed in English taking into consideration Health Belief Model (HBM) constructs and brainstorming among research team members on what topics to focus on in order to achieve the main objective of the study. Prior data collection, FGD guide was translated into local language (Kinyarwanda) by one of the research team members (JBN) and was checked against its English version by other research team members (JPN, SN and GU) who know Kinyarwanda.

Participants were first asked general questions exploring common or well-known diseases in the local community and the knowledge about the heart and its function in the human body. They were asked questions about what they think can harm their heart; about how they perceive the possibility of being exposed to things that can affect their heart; and about how they can explain heart disease. FGD participants were also asked about their health seeking actions in the prior 12 months, what makes them believe that a disease is very harmful and the things that are in their local community that can cause general ill-health. They were also asked to cite what might cause harm or danger to their heart in daily life, and how information around the possibility or likelihood of having a disease or dying from disease had been communicated in their communities. They were also asked how they would want the message on having a heart related disease, or the likelihood of dying of a heart related disease to be communicated to them. The ability of FGD participants to understand their disease risk compared to others was assessed by asking them the following question: “If a doctor tells you that the likelihood of you having a particular disease e.g. is five or more times than that of your friend or neighbour of the same age, how would you understand or interpret this statement from the Doctor.” Lastly, FGD participants were given narrative and visual (thermometer and bar chart) formats indicating different levels of risk (risk score) and asked to interpret them (for more details, see FGD guide in Appendix 1). Each FGD was moderated by two researchers who were part of the investigators team and experienced in FGD facilitation; JBN served as the facilitator whereas JPN or GU served as notes taker. FGD lasted between 1h45min and 2h46min and data collection took place during the period of September-November 2019.

At the time of FGD, the study participants were compensated for their cost of travelling to the site of FGD and each FGD participant was given 5,000 Rwandan francs (equivalent to 4.96 USD).

### Analysis

Data were analyzed using thematic analysis following the six steps described by Vaismoradi, et al. [12]: (i) familiarizing with the data; (ii) generating initial codes; (iii) searching for themes; (iv) reviewing themes; (v) defining and naming themes; and (vi) producing the report. Even though the FGD topic guide was conceived using HBM constructs, an inductive approach was used during analysis in order to allow unexpected themes to merge. The analysis was performed using Atlas.ti 7.5.18.

Prior to analysis, eight FGD recordings were transcribed verbatim in the local language (Kinyarwanda) and translated into English. After transcription, JBN checked each transcript against its original audio. After translation, JBN also verified transcript English version against its Kinyarwanda version.

Analysis was done by three researchers (JBN, GU & JPN), who all read the first FGD transcript of the rural study site. To ensure inter-rater consistency, these researchers met to compare individual codes and resolve discrepancies. All three researchers continued independent analysis using the second transcript of the FGD conducted in the urban study site. Newly identified codes and themes were discussed in another meeting. Using the codes agreed upon during this meeting, one researcher / analyst (JBN) continued analysis of the six remaining FGDs. JBN then shared the updated thematic codebook with GU and JPN for discussion until consensus was reached. Afterwards, JBN continued with the phases of reviewing themes, defining and naming themes, and producing the final analysis report.

Results were reported as a narrative summary of main findings for each theme and one or two selected quotes from FGD participants.

## Findings

Overall, 65 out of 77 recruited potential participants attended their allocated FGDs. In other words, 12 (16%) recruited potential participants did not show up at their respective venues designed for FGD. The size of FGD varied between 6 and 12, the lowest attendance rate was among the FGDs with youth from each study site. The median age (in years) of study participants was 29 (22-39), a little more than half were women (*n*=34; 52.3%), and more than half were adult (*n*=39; 60%). The Table 1 below indicates details on demographic characteristics of study participants:

**Table 1:** Demographic characteristics of the study participants

Variable	Total (65)	Men, 47% (31)	Women, 52.3% (34)	Youth (18–24), 40% (26)	Adult (25–65), 60% (39)
**Age in years, median (P25-P75)**	29 (22–39)	29 (22–39)	28.5 (22–40)	21.5 (19–22)	36 (30–47)
**Residence, % (n)**					
Rural	52.3% (34)	58.1% (18)	47.1% (16)	53.8 (14)	51.3% (20)
Urban	47.7% (31)	41.9% (13)	52.9% (18)	46.2 (12)	48.7% (19)
**Education, % (n)**					
Not completed primary Education	17.0% (11)	19.3% (6)	14.7% (5)	15.4% (4)	17.9% (7)
Completed primary Education	29.2% (19)	32.3% (10)	26.5% (9)	7.7% (2)	43.6% (17)
Not completed Secondary education^a^	52.3%(34)	45.2 (14)	58.8% (20)	76.9% (20)	35.9% (14)
Completed secondary education	1.5% (1)	3.2% (1)	0.0% (0)	0.0% (0)	2.6% (1)
**Religion, % (n)**					
Roman Catholic	35.4% (23)	38.7% (12)	32.3% (11)	30.8% (8)	38.5% (15)
Pentecostal	18.5% (12)	16.2% (3)	20.6% (7)	26.9% (7)	12.8% (5)
Protestant	10.8 (7)	6.5% (2)	14.7 (5)	11.5% (3)	10.3% (4)
Jehovah’s Witness	1.5% (1)	3.2% (1)	0.0% (0)	0.0% (0)	2.5% (1)
Seven-day Adventist	27.7% (18)	29.0% (9)	26.5 (9)	23.1% (6)	30.8% (12)
Muslim	1.5% (1)	3.2% (1)	0.0% (0)	3.9% (1)	0.0% (0)
Other^b^	4.6% (3)	3.2% (1)	5.9% (2)	3.9% (1)	5.1% (2)
**Occupation, % (n)**					
Cultivator	41.5% (27)	41.9% (13)	41.2% (14)	26.9% (7)	51.3% (20)
Retailer	9.2% (6)	9.7% (3)	8.8% (3)	3.8% (1)	12.8% (5)
Labourer	7.7% (5)	16.1% (5)	0.0% (0)	3.8% (1)	10.2% (4)
Tailor	4.6% (3)	0.0% (0)	8.8% (3)	7.7% (2)	2.6% (1)
Security Agent	1.5% (1)	3.2% (1)	0.0% (1)	0.0% (0)	2.6% (1)
Student	13.9% (9)	25.1% (8)	2.9% (1)	34.6% (9)	0.0% (0)
Unemployed	21.6% (14)	3.2% (1)	38.3% (13)	23.1% (6)	20.5% (8)

^a^ Include those who are still students and those who had left school, ^b^ Either Restoration church, God’s Good Mission church or Apostolic Church

The findings are presented in the form of themes that emerged from the FGDs and are pertaining to CVD risk communication. These themes and their sub-themes are provided in Table 2 below:

**Table 2:** Themes and sub-themes

Number	Themes	Sub-themes
1	Perception and representation of disease related harm	Chronicity of a disease, being selective for foods and drinks, diseases leading to sudden death, impediment to physiological functions, fatality if no medicine taken, disease symptoms and length of hospital stay
2	Perceived risk of CVD	Financial stress, psychosocial stress, substance abuse, noise pollution, unhealthy diet, diabetes and overworking
3	Free and voluntary screening for CVD risk factors	
4	Existing ways of communicating disease risk	Outreach campaign, lack of communication of a disease risk at health facility level
5	Preferences of CVD risk screening and communication	Using mobile health technology for CVD (risk) screening, CVD risk screening by community health workers (CHWs), preferred ways of communicating the likelihood of a disease
6	Perception towards counseling services at health facility	Inadequate counseling services at health facility, unawareness of existing NCD (CVD) services at health facility
7	Interpretation or understanding of the (CVD) risk presentation formats	

### Perception and representation of disease related harm

Across all FGDs, chronicity of a disease, being selective to foods and drinks, diseases leading to sudden death, disease that impedes some physiological functions, attack rate, fatality if no medicine taken, disease symptoms and length of hospital stay are factors which were perceived as being linked to a harmful disease. 

**Fig. 1:**
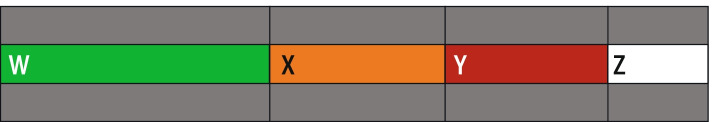
Colours that may be used to represent risk

During this study, we assessed which colour represents a high risk by giving each participant a piece of paper with four different colours (red, yellow, green and white), as indicated in Fig. 1, and asking them to identify one colour that represents a high risk. Red colour was perceived by almost all study participants to be related to the highest risk.

With the same piece of paper, FGD participants were also asked to choose one colour they think can be used to represent the least (or smallest) possible harm. Across all FGDs, participants reported that either orange or green colours may be used to represent the lowest risk. However, some participants mentioned that white can also be used to represent the least (or smallest) possible harm.

### Perceived risk of CVD

When participants were asked about their perceived risk of suffering from CVD, those who perceived to be at risk attributed this to either financial stress, psychosocial stress, substance abuse, noise pollution, unhealthy diet, diabetes, or overworking.

### Financial stress

Across FGDs, participants expressed that they are likely to suffer from CVD because of financial constraint. Financial constraint was perceived as either failure to sustain a small business, incapacity to feed their family, bankruptcy, or poverty in general.

“Most of the people do not have income generating activities and those who try to venture into small business, they are not successful. In this life condition, people think much especially how to pay school fees for children or foodstuff which leads to living a misery life and consequently get attacked by various diseases including heart attack.”: Participant #2, FGD #4 (Adult women), Urban site

“Poverty causes headache and heart disease.” (Participant #2, FGD #2 (Adult women), Rural site

### Psychosocial stress

In both study sites, FDG participants perceived themselves to be at CVD risk due to psychosocial stress. This was expressed as, e.g., deception in romantic relationships among young people, gender-based violence within couples, violence committed to children (heavily beaten by their parents), individual reactions towards a stressful situation (when they are told of or witness tragic events, they are told off etc.), being gossiped, experiencing (or being told about) a tragic event, anxiety, overthinking daily life and depression.

“It’s what they were talking about the husband; constant worries and stress caused by your husband at the point you feel like you are about to lose your mind. Another thing is a tragic event when you lose your relative, it might cause you harm or danger to your heart.”: Participant #9, FGD #2 (Adult women), Rural site

“Another thing, in our region parents like to beat their children, so you may find a child is always afraid and traumatized which can lead him/her to have a heart disease.”: Participant # 1 (Young men), Urban site

### Substance abuse

Young men from both communities, and adult men from the urban community perceived themselves to be at risk from CVD because of the consumption of alcoholic beverages. Most participants who reported alcohol consumption also smoked cigarettes and perceived themselves to be at risk of CVD. Only adult and young men who self-reported smoking cigarettes or illicit drugs (like marijuana) which put them at CVD risk.

“For me, three things can cause troubles to my heart. First of all, I smoke cigarette. Secondly, I drink beer and thirdly, I think so much such that my heart never get rest.”: Participant #5, FGD #3 (Adult men), Urban site

One adult woman from the urban community perceived herself to be at risk of suffering from CVDs because of secondhand smoke produced by marijuana smoked by young people in their local community.

“For me, drugs can also cause trouble to our hearts especially for youth and generally for our children. When it comes to the evening, young people lock themselves and smoke marijuana, and smell is widespread to non-smokers and affect them without leaving the smokers behind.”: Participant #4, FGD #4 (Adult women), Urban site

### Noise pollution

Across FGDs, some participants claimed to be exposed to environmental noise which may put them at risk of CVD. The sources of environmental noise were bars, churches, loud music played on the radio or factories found in their neighborhood (like cassava flour making machines).

“Thank you. We are living with young people who play loud music in the mid night. I think that this noise can cause troubles to our heart in the future...” Participant #3, FGD #4 (Adult women), Urban site

### Unhealthy diet

In both study sites, FGD participants perceived that they are likely to suffer from CVD because of unhealthy diets. Unhealthy diet was expressed as eating either industrial / processed food, meat, too much oil or too much salt. One adult woman in the FGD conducted in the urban community attributed their habit of consuming unhealthy food to poverty, hindering them to afford a healthy diet.

“I also think that I may get a heart attack in the near future because of industrial food that contain toxics. Eating cow’s meat or oil may also cause heart-related diseases. Healthy cooking oil are not affordable to poor people.”: Participant #11, FGD #4 (Adult women), Urban site

In the urban community, women (both young and adult) revealed that they eat food based on deliciousness and not on their nutritious function.

“… We are also eating food based on deliciousness not on the nutritious importance. We seem to ignore what are the negative consequences that may occur in the future. Certainly, I may get heart attack in the future.”: Participant #3, FGD #4 (Adult women), Urban site

### Diabetes

One young man in the urban site asserted that he perceived himself to be at CVD risk because he was diagnosed with diabetes risk.

“Last year I went to the hospital and the doctor told me to stop taking much sugar because I was at risk of having diabetes, and diabetes can lead to heart diseases.”: Participant #4, FGD #7 (Young men), Urban site

### Overworking

Adult study participants from the urban community reported that they may suffer from CVD because of overworking in order to satisfy their needs.

“For instance, I went to hospital last week and after testing me they told me that I have to take enough rest in order to allow my heart function well. But I find it is difficult to rest because I have to work tirelessly in order to get money and satisfy my needs.”: Participant #4, FGD #3 (Adult men), Urban site

### Free and voluntary screening for CVD risk factors

Only one participant in the FGD with men from the urban community reported to be aware of the car free day promoting mass sports in which the Rwanda Ministry of Health and Development partners avail medical staff and kits to screen car free day participants for CVD risk factors like hypertension and diabetes for free.

**“**…there is a chance to get tested these NCDs for free of charge during the “Sports for All” an event organized by the MoH that is held once a month. Some people feared to go to hospital due to lack of financial means but now it is high time to participate in that “sports for all” so as to have a chance to get tested and know their health status.”: Participant, FGD #3 (Adult men), Urban site

Only in the FGD with adult women in the urban community, two participants revealed that they voluntarily go to seek CVD risk factor screening services from their health facility. One of these participants reported to be surprised for being diagnosed with a CVD risk factor (hypertension):

“What I can tell you is that even last week I was sick. When I went to hospital for medical check-up, the test results showed that I have blood pressure which is obviously related to heart disease. I could not imagine that I can have such blood pressure and it was high blood pressure...”: Participant #2, FGD #4 (Adult women), Urban site

Although some FGD participants were aware of the opportunity of accessing free CVD (risk) screening, other FGD participants were not aware of this opportunity:

“From what I know, not everyone has been mobilized to regularly go for medical check-up so to know his/her health status in order to take the preventive measures. It is possible that someone can spend more than five years without going to the hospital to know his / her health status and that is why some community members die abruptly because of disease they did not know before. People do not have the same level of understanding where one can advise herself to go for medical check-up.”: Participant #9, FGD #4 (Adult women), Urban site

### Existing ways of communicating disease risk

In both study sites, we found that disease awareness in the community is conducted in the form of meetings, whereby health professionals provide information about disease outbreaks (e.g. increase of malaria cases) or a new disease for which community members are at risk (e.g. Ebola). In these outreach campaigns, health professionals teach the community about symptoms, causes, mode of transmission, prevention of the concerned disease, and sometimes screen participants for that disease and treat positive cases. It is important to note that, across all FGDs, participants did not mention NCDs being discussed in community meetings.

“If there is an emerging disease where health care providers will notice a sudden increase of patients suffering from that disease, like malaria for example. In that case, the health centre organizes an outreach program where they come in the community and teach how to prevent that disease and test us on site for that disease.”: Participant, FGD #1 (Adult men), Rural site

Communication of the likelihood of getting a disease is almost non-existent. Across all FGDs, only one participant asserted that a doctor can tell a person that he has the likelihood of getting a disease:

“…the doctor may tell you that they diagnosed diabetes as an example; or they tell you that you are about to get this disease, and maybe the medicines they give you, should reduce the severity; or they just put you on a diet.”: Participant #6, FGD #6 (Young women), Rural site

### Preferences on CVD risk screening and communication

FGD participants suggested using technology in screening for CVD risk, for example by using (smart) phones. In each FGD conducted in urban community, at least one participant suggested that community health workers (CHWs) be trained on the use of mobile health (m-Health) technology to conduct door-to-door CVD (risk) screening and provide medications:

“I have an idea, it would be better if CHWs have that system and pass to every family, for example if they have that machine that check and give immediate results and medicaments.”: Participant, FGD #7 (Young men), Urban site

Across FGDs, most participants preferred to be told about the likelihood of a disease in the form of (private) conversation or counseling:

“I wish my doctor / health care provider to give me a message in a way of counseling. I would like him / her to talk to me privately and advise me what to do, how to behave but not everyone to know my case.”: Participant, FGD #3 (Adult men), Urban site

**“**…. S/he can start the discussion with simple things, and continue telling you about that disease, how can someone contract it, what can cause it and its symptoms. He can finish the discussion by telling you that you have the likelihood of having that disease in the future.”: Participant, FGD #8 (Adult men), Rural site

Only young men from the rural community FGD suggested to present the likelihood of a disease happening to a person using colours. Some of them suggested to use the red colour to present high risk; yellow the moderate risk and green the absence of or low risk:

“…. For example, if you use a red colour, you explain that it represents someone at the highest level, a yellow colour represents someone who can be treated and cured; green colour someone who is safe.”: Participant #6, FGD #5 (Young men), Rural site

### Perception towards counseling services at the health facility

FGD participants reported not being provided with adequate counseling services at the health facility. They reported that there are some health services providers who poorly communicate with patients. This was indicated by saying e.g., that health providers do not share information about their disease after conducting diagnostic tests; when they talk to them they do so in unfriendly ways, as per the quotes below:

“… My wife and I go to the hospital more often but whenever we go there, we explain to the health care provider the way we feel and the latter give only medicine without telling us what we are suffering from.”: Participant #2, FGD #1 (Adult men), Rural site

“I think that s/he can talk to you in a good way advising you to stop anything bad to your health because there are some who talk so badly with pressure and stressing you which is not good, but when s/he talks to you kindly you can follow what you are ordered and have a good follow up.”: Participant #5, FGD #8 (Young women), Urban site

In the rural community, study participants suggested having one health provider specialized or trained in CVDs who can provide CVD services. This expressed need can be perceived as an indicator that community members are not aware of, or sensitized on NCDs services provided at the HC as the selection of study sites was based on the criteria of a community (HC catchment area) served by a HC providing NCDs services and having a nurse in charge of NCDs (including CVD) services.

**“**Health centers don’t have doctors / health services providers who treat heart disease, it could be better if we have one at the health center specialized only on that.”: Participant, FGD #1 (Adult men), Rural site

### Interpretation or understanding of (CVD) risk presentation

**Fig. 2:**
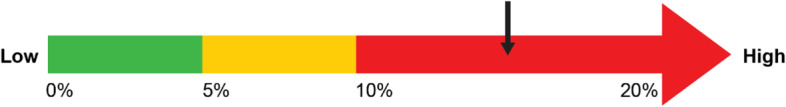
Risk shown in a thermometer without explanation text

In the context of testing the ability of community members to interpret disease risk presented in the form of either a thermometer or a bar graph whereby each FGD participant was given a piece of paper displaying samples of risk presentation, and asked: “If the health services provider gives you your medical test results in the form of that piece of paper, does it help you to appreciate or evaluate your likelihood of having a disease?” Most participants provided irrelevant answers. Findings from each risk presentation format are described below:

Thermometer without label on the arrow indicating the level of likelihood of having a disease in the next five years (Fig. 2). The correct interpretation would be stated as follow: “If a health care provider gives me my medical test results in the form of that piece of paper, I will interpret it as I have a 10% chance or more of developing the concerned disease.” Alternatively, the right answer may be stated as: “If a health care provider gives me my medical test results in the form of that piece of paper, I will interpret it as I have a 10 in 100 chance or more (or 1 in 10 chance or more) of developing the concerned disease.” Across all FGDs, only one participant from the FGD with young men in the urban community provided the right answer.

**Fig. 3:**
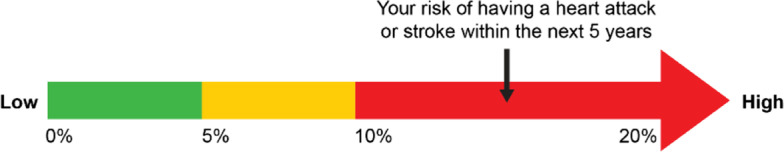
Risk shown in a thermometer with explanation text

Thermometer with label on the arrow indicating the level of likelihood of having a disease in the next five years (Fig. 3 which should be interpreted in the same as Fig. 2): across all FGDs no participant was able to correctly state the message conveyed by this risk presentation format.

**Fig. 4:**
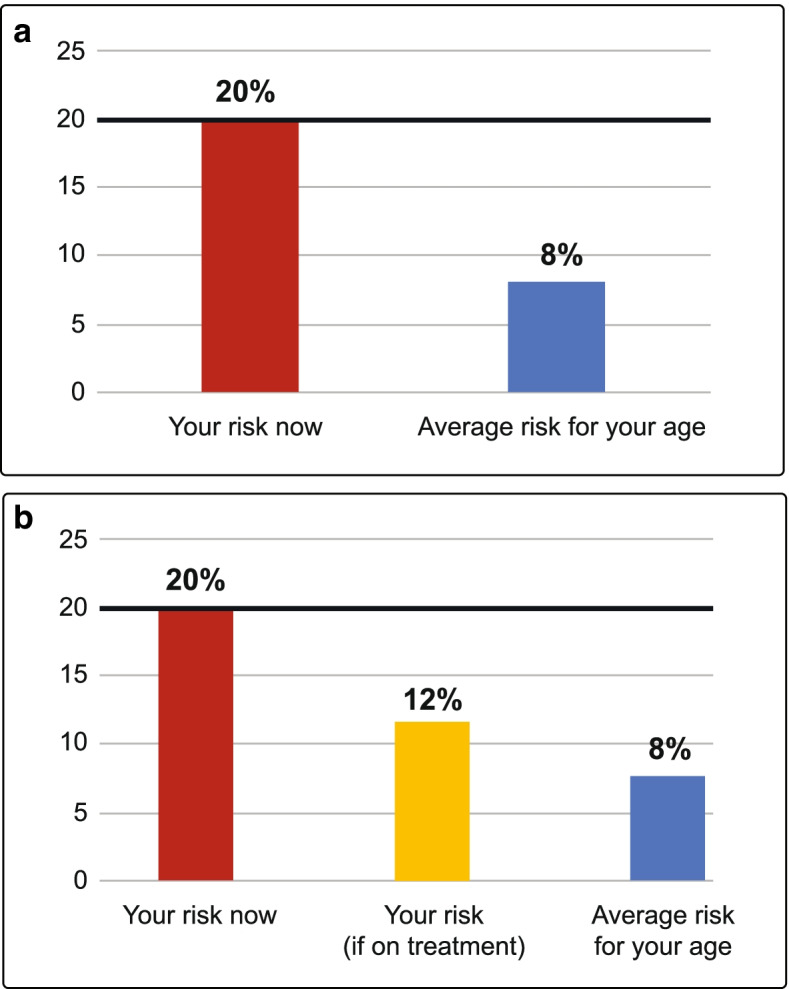
**a** CVD risk presented in bar graph format. **b** CVD risk presented in bar graph format

Bar graphs with a label under each (rectangular) bar (Fig. 4a & b): only a few participants from the FGD with adult men in the rural community and the FGD with young women in the urban community were able to correctly state the message conveyed by this risk presentation format.

Overall, we found that community members are not able to interpret / understand disease risk, neither when presented in thermometer format nor in bar graph format. FGD participants indicated that they do not understand these formats because of their low level of education. Some suggested explaining or communicating disease risk verbally (in person communication) instead of giving them a piece of paper and requesting them to interpret it.

“This method is not understandable 100% because not everyone can read it and get the meaning of his / her results, according to the capacity of the country not everyone has education, some have it, others don’t. Not every person can explain him/herself that, so that’s why I say that not everyone can know his / her results of the problem he has.”: Participant #1, FGD #7 (Young men), Urban site.

When assessing the ability of FGD participants to understand their disease risk compared to others, they were asked the following question: “If a doctor tells you that the likelihood of you having a particular disease e.g. is five or more times than that of your friend or neighbour of the same age, how would you understand or interpret this statement from the Doctor?” FGD participants did not understand the question and some FGD participants were only able to correctly interpret / answer the question after it was rephrased by the researcher.

“If he tells me that I have five times chance of having particular disease more than my friend, I would think that I’m about to get seriously sick.”: Participant #8, FGD #1(Adult men), Rural site.

“I think that, one is more exposed to the risk of getting that disease and get impacts than me.”: Participant #5, FGD #7 (Young men), Urban site.

Despite repeated efforts made by the researcher to rephrase and contextualize the question, some FGD participants were unable to understand the sentence, and some participants declared that they didn’t understand the sentence at all, gave incorrect or vague or strange answers, and confused disease risk with the actual disease, as illustrated in the quotes below:

“If the doctor gives me an example that my brother’s vulnerability is five times than mine. I can take the signs my brother has and multiply them by 5% in order to know the degree of my vulnerability. From there, I can draw a conclusion that based on that %, I am going to respect doctor’s advice in order to reduce the degree of vulnerability.”: Participant #1, FGD #4(Adult women), Urban site.

“We do not really understand.”: Participant #7, FGD #6 (Young women), Rural site.

One participant from the urban FGD with young women provided an interpretation opposing the real message conveyed by the statement:

“I think that s/he is trying to tell me that my friend is very sick.”: Participant, FGD #8 (Young women), Urban site.

When asked about alternative ways to present disease risk, FGD participants in the rural community suggested using colours like yellow, green and red in order to represent different levels of risk:

“My suggestion is that on the drawing you used, there can be another way of making them more understandable; e.g. the colours you used: yellow, green and red, you could have written something like a label on each of the colour indicating what they mean. That’s my suggestion.”: Participant #2, FGD #1 (Adult men), Rural site.

## Discussion

This study aimed at providing health services stakeholders and decision makers with information on the understanding of, and perception towards CVD risk and CVD risk communication among rural and urban community members with low educational attainment in Rwanda. It was found that study participants perceived themselves to be at risk of CVD because of financial stress, psychosocial stress, alcohol consumption, substance abuse, noise pollution, unhealthy diet, diabetes and overworking. Although there are opportunities of CVD risk factors screening free of charge, through for example car free day mass sports, study participants were not aware of this. This study also revealed that counseling services at health facilities were judged to be poor.

Importantly, this study revealed that CVD risk communication services do not exist in health facilities that were part of our study sites. It was found that study participants have inadequate health literacy to understand and interpret CVD risk as presented in a thermometer or bar graph format and preferred receiving CVD risk communication through in-person or verbal communication. As regards CVD risk screening, study participants also preferred training of CHWs to screen for CVD risk and using mobile phone technology. The culture of seeking CVD preventive healthcare services among study participants was quasi-inexistent, which is not in line with another study conducted in developed country, United States of America, where individuals attended health facilities for CVD preventive health care [13].

Factors perceived by study participants to lead to CVD risk were not different from findings of studies previously conducted in other contexts, including African countries. Stress which was perceived by most study participants to be a risk factor for CVD, was also reported by study participants in Kenya [14], Nigeria [15], South Africa [10] and America [16]. Moreover, stress was cited as the most perceived cause of heart disease in a recent qualitative meta-synthesis on CVD [17]. Stress in this study was linked to financial constraint to afford basic needs, similar to a report of perceptions of heart-healthy behaviour among African American adults [16]. Stress was also linked to psychosocial stress related to gender based violence, which was also reported in a qualitative study conducted in Kenya [14]. The finding that some participants from this study, and particularly women from the rural community, perceived themselves to be at risk of CVDs because of witnessing or being told about tragic news or being told off, is in line with another study conducted in South Africa where respondents thought that hearing tragic news can cause strokes [10].

Importantly, the finding that young people in this study perceived themselves to be at CVD risk seems not surprising as cases of hemorrhagic stroke have already been reported among young adolescents in Rwanda [18]. This indicates a need for population-based interventions targeting CVD prevention in all age groups.

Poverty was indirectly perceived as a source of financial stress, which is in line with the findings from another study conducted in Kenya where poverty was considered as an underlying risk factor for CVD [14]. In addition, in this study, poverty was linked to unhealthy diets which also led study participants to feel at risk of CVDs, in line with a study conducted by Surka et al. [10], in which respondents associated poverty with unhealthy diets and felt at risk of CVDs because of this. In Nigeria, adolescents and young adults perceived that unhealthy (poor) diet could put them at CVD risk [15]. Unhealthy diet in this study was linked to eating industrial / processed food, meat, too much oil or too much salt. These findings are in line with findings from other studies that investigated healthy individuals’ self-perception towards CVD risk in Kenya [10] and South Africa [14]. Unhealthy food were also attributed to poverty, whereby some community members would consume unhealthy food out of convenience, findings that are in agreement with another study conducted in America [16]. Some participants in the FGD with women reported to consume unhealthy food because of its deliciousness, which is in line with findings from a study recently conducted in another African context where women reported difficulties to avoid preparing fatty food because of the pressure to satisfy the tastes of their husbands [14]. This indicates that, in some African contexts, cultural beliefs can be perceived as contributing factors to CVD risk exposure.

CVD risk in this study was also linked to excessive or harmful alcohol consumption and tobacco. Some study participants who felt at risk of CVD due to alcohol consumption advanced stress as a pretext to engage in this unhealthy behaviour, an argument also recorded in a study conducted in Kenya [14]. Other participants in this study did not acknowledge to be at CVD risk due to substance abuse because of lack of knowledge, which was also found in a study conducted in Kenya, where it was reported that most study participants were not aware of the association between smoking and excessive harmful alcohol consumption and CVD [14].

Environmental pollution, in the form of flour particulates produced by cassava flour making machines, was reported in this study as a risk factor for CVDs in the urban community, which was also found in another study on CVD risk factors in urban informal settings in Kenya [14]. In the latter study, dwellers reported that kerosene, wood charcoal for cooking, gaseous emissions and effluents from industries expose them to health risk [14].

Lack of awareness around opportunities to screen for CVD risk factors through mass sports, such as car free day sport, could be perceived as a proxy indicator of ineffective sensitization around CVD prevention which, in turn, could be translated into weak primary CVD prevention. Study participants in this study were not aware of CVD screening and treatment services at their nearest health facilities (health centers), which is in line with a study which reported the need to improve the quality of primary prevention for CVD in LMICs [19]. The suggestion / preference of study participants to train CHWs on CVD screening and to conduct door-to-door CVD screening were also recorded in another study in the region, where study participants suggested that community health volunteers can be used to conduct CVD screening in the community [14]. Moreover, in this study, FGD participants suggested using mobile health technology to screen individuals for CVD risk. This concurs with the study in Kenya where short messaging service and the use of social media platforms were suggested as strategies to disseminate messages on CVD risk, available opportunities to screen, and advice on where care and treatment could be sought [14]. One of the supporting ideas for involving CHWs in CVD primary prevention is the community members awareness on CHWs’ success in other areas of health services provision, which was also found in the study in Kenya [14].

Being able to understand risk is only one of the factors that influence how people understand and use information in their decision-making [10]. Unfortunately, in this study, participants were unable to understand and interpret different CVD risk presentation formats, including thermometer and bar graph formats indicating the probability of a CVD event within five years. This inability to understand and interpret CVD risk was also noticed in a study recently conducted in South Africa [10], whereas both thermometer and bar graph formats were understood and preferred among patients and general practitioners in Australia [20]. The inability to understand and interpret CVD risk was also found in another study where the presentation of CVD risk using quantitative risk formats was only understood by highly educated individuals [21].

In this study, in-person conversation or counseling was suggested as a means to communicate CVD risk, which was also in line with findings from the study conducted in Australia, where qualitative formats were preferred over quantitative formats [21]. In the present study, participants reported to not understand quantitative formats because of their low level of education, which is not surprising as many people find it difficult to comprehend basic probabilities [11]. However, individual characteristics such as age, education and numeracy, may also affect risk comprehension and its qualitative interpretation [22].

A major strength of this study is that it considered both urban and rural areas, and youth and adults. The study was limited to community members with a low level of education and does not reflect the risk perception of individuals with higher levels of education (i.e., who attended at least university or any other tertiary level of education). However, it was reasonable to conduct this study among individuals with a low level of education since the proportion of individuals who attended university in Rwanda is 1.9% [23].

This qualitative study provides useful information to policy makers and programme implementers in designing programmes to enhance awareness on CVD risk and risk factors and insights into how to deliver effective CVD prevention services and care, particularly in Rwanda and in low-resourced communities in general.

## Conclusion

This study showed that some rural and urban community members, in Rwanda, are aware of what could put them at CVD risk in their respective local communities; however, others are not be aware of what could put them at CVD risk because of their lack of knowledge of CVD (risk factors). This study also showed that quantitative formats to present CVD risk are less suitable within the context of Rwanda. As a result, we recommend using qualitative formats (low risk, moderate risk, and high risk) to present CVD risk. If quantitative formats are used, this should be accompanied by qualitative interpretation (and counselling sessions) from a health professional. Community based-CVD risk screening conducted by CHWs are likely to be accepted by local community dwellers. The use of colours could also be a possible way to represent CVD risk. This qualitative study provided useful information to policy makers and programme implementers in designing programmes to enhance awareness on CVD risk and risk factors and insights into how to deliver effective CVD prevention and care, particularly in Rwanda and in low-resourced communities in general. Action research about the capacities of CHWs to screen their fellow community members for CVD risk using mobile heath technology is hence recommended. Operational research on the use of qualitative formats to communicate CVD risk is recommended to improve decision-making on CVD risk communication in the context of Rwanda as well.

## Supplementary Information

**Additional file 1. **

## Data Availability

To be compliant with the health leading institutions' research and data sharing policy in place, the raw data analyzed during this study are available from the corresponding author on reasonable request.

## Abbreviations

CHWs: Community health workers
CVD: Cardiovascular disease
HBM: Health belief model
HC: Health centre
HICs: High-income countries
FGDs: Focus Group Discussions
LMICs: Low- and middle- income countries
MoH: Ministry of Health
NCDs: Non-communicable diseases
RNEC: Rwanda National Ethics Committee
STEPS: STEPwise approach to surveillance
WHO: Word Health Organization
