# Supplementary material for: Perceived cardiovascular disease risk and tailored communication strategies among rural and urban community dwellers in Rwanda: a qualitative study
Source: BMC Public Health. 2022 May 9;22:920. doi: 10.1186/s12889-022-13330-6 (PMC9088034; doi:10.1186/s12889-022-13330-6)
Supplement: Supplementary file 1 — Additional file 1. [file 12889_2022_13330_MOESM1_ESM.pdf]

## APPENDIX 1: FOCUS GROUP DISCUSSION GUIDE

### Introduction

**Questions to guide the discussion:**

**General:**

i) What do you think are the diseases that you consider harmful or common in these communities? Probe for NCDs – diabetes, cancer, heart diseases, hypertension, stroke, etc.

ii) We would like to know what do people in these community including you, think or know about the heart. Do please let us know the following:

- What does the heart do, and how important is the heart to you?

**Perceived threat** (Perceived Threat/Vulnerability)

iii) What are the specific things you think can harm the health of your heart? Please explain how these things can affect the heart

iv) Do you perceive or feel any possibility of having or being exposed to things that may affect your health, and possibly your heart?

v) Please explain what you (and people in your community) understand *heart disease* to be (Tips: *How many of us here have an idea of what heart disease/heart-related diseases are? Tell us more*).

vi) Do you personally believe that there is a chance that you might get heart disease in the future?

**Health seeking actions (Behaviour intentions)**

vii) In the past 12 months, how often have you attended (or taken a relative to) a clinic/hospital/doctor's room/ alternative/ traditional healer place for your health? Where have you gone and for what problem?
*Probe to see if visited traditional or medical/health practitioners for heart-related event.*

**Concept of Risk**

viii) What are the things that make you (or others) to feel or believe that a particular disease (or an event) can be **very harmful**?

ix) What are things you think that **you and others** are exposed to(or involved in everyday life)in your community that 1) can be harmful to you, and, 2) maycause **you**general ill health?

x) What things in your community and everyday life do you think might cause harm or danger to your heart in particular? *Probe on risk factors (smoking, alcohol, diabetes, hypertension, weight gain, environmental, SES). Also ask about psychological factors - anxiety, depression, stress, loss of a relative or loved one, etc).*

**Communication and presentation of risk**

xi) Could you think of a way that information on the possibility or 'likelihood' anyone having a disease or dying from a disease has been communicated to you or your community (may be by your mother, teacher, doctor, nurse/CHWs, friends, class-mates, etc)?
*Probe to see if there is any form of communication around CVD risk in the community*

xii) How would you want the message on the possibility of having or the likelihood of dying of a heart-related disease (e.g. heart disease, stroke, angina, or hypertension) to be communicated to you for your proper understanding?

xiii) If a doctor tells you that the likelihood of you having a particular disease e.g. is 5 or more times than that of your friend or neighbour of the same age, how would you understand or interpret this statement from the Doctor?

xiv) Are there any other issues or questions you would like to discuss related to this topic that I have not asked?

Now go to the **'Cue Cards'** for risk communication. Each participant to answer these questions.

***Task setting:*** This is intended to be used to set specific task (steps) for Citizen Science interview in the context of each setting. E.g. the things mentioned here would be those we would ask the CSts to specifically capture (narrative and pictures) in the field.

## B: RISK COMMUNICATION - CUE CARDS

<table>
<tr><th>Key focus</th><th colspan="4">Risk Communication Semi-Structured interviews with FGD Participants</th></tr>
<tr><td><u>Concept of risk</u></td><td colspan="4">Q1: Which of these colours in your mind, would you say can be used to represent a very high possible harm or danger sign, or threat to life, or death?<br>If none, say so, and do let us know the colour you think may be used to represent a very high risk.</td></tr>
<tr><td>Answer:<br>Point to the colour(s)</td><td>w</td><td>x</td><td>y</td><td>z</td></tr>
<tr><td></td><td colspan="4">Q2: In your mind, which of these colours below signifies the least (or smallest) possible harm or threat of death, or danger? If none, say so.<br>If none, say so, do let us know the colour you think may be used to represent a smallest(or very least) harm or risk.</td></tr>
<tr><td>Answer:<br>Point to the colour(s)</td><td>w</td><td>x</td><td>y</td><td>z</td></tr>
<tr><td></td><td colspan="4">Is there is any other colour apart from these that you think can represent high possibility of harm, danger or disease? Please, tell us more.</td></tr>
<tr><td rowspan="2">Presentation and <u>communication of risk</u></td><td colspan="4">Q3: How do people (your parents, friends, doctor, nurse, etc) present the likelihood of someone having a disease or the likelihood to die of a sickness or an event in your community?</td></tr>
<tr><td colspan="4">Q4:How would you want the message on the possibility of having a certain level of likelihood to die of a diseases (CVD) for your understanding?</td></tr>
<tr><td><u>Comprehension</u> and Preference of risk communication format</td><td colspan="4">Q5: If a doctor or nurse (or any health worker) shows you these options (Options 1-3) to help you to understand the possibility of you having a disease (such as hypertension, heart attack, heart failure,  or stroke, etc) in the next 5years.<br>Tell us, in your opinion, how does each of these (risk) presentation options helps you to appreciate or evaluate the likelihood of having the said disease?Please Ask in succession</td></tr>
<tr><td rowspan="2">Risk shown in a<br><u>thermometer</u><br><br>(Option 1)</td><td colspan="4">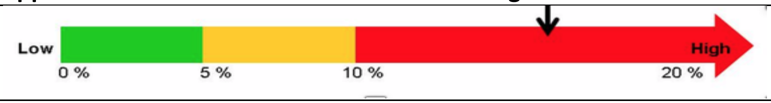
</td></tr>
<tr><td colspan="4">Could you please describe in your own way what you understand about this risk presentation (Option 1): How does it helps you to appreciate or evaluate your likelihood of having a disease?</td></tr>
<tr><td>Risk shown in<br>a<u>thermometer</u><br>(with explanations)<br>(Option 2)</td><td colspan="4">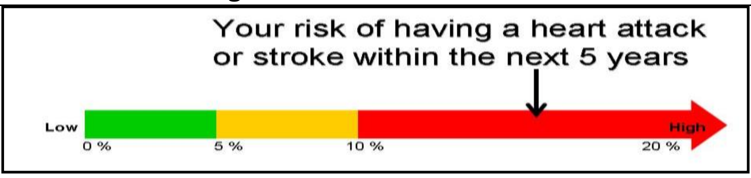
</td></tr>
<tr><td></td><td colspan="4">Could you please describe in your own way what you understand about this risk presentation (Option 2): How does it helps you to appreciate or evaluate your likelihood of having a disease?</td></tr>
</table>

| | |
|---|---|
| Risk shown in **a Bar Graph**<br><br>With legends **(Option 3a & b)** | 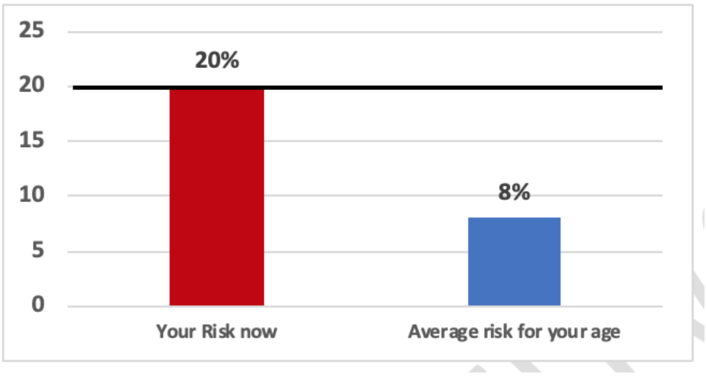<br>**Option 3a**<br>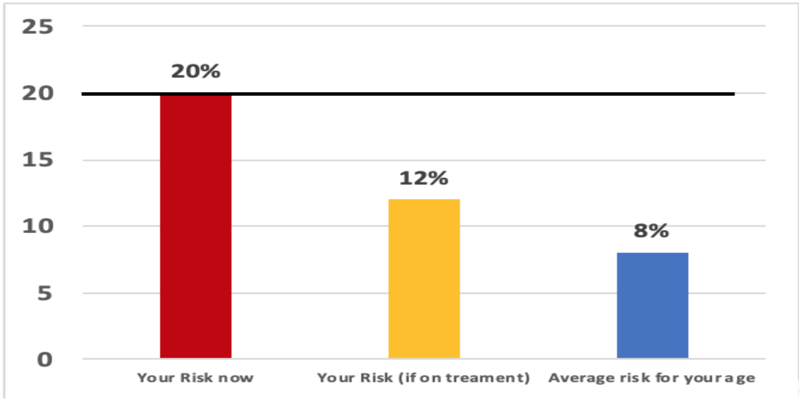<br>**Option 3b** |
| | Could you please describe in your own way what you understand about these risk presentations (Option 3a & b): How does it **helps you to appreciate or evaluate your likelihood of having a disease?** |
| | Can you say, you have understood the presentations in Options 1-3? Which one would you prefer amongst them? |
| | Print each option (1-3) in a separate cardboard paper or an A4 paper |
| Your own risk (**Option 4**) | Could you describe or make a sketch of any way (risk presentation option) thatyou think would make more sense to you about a likelihood of someone having a disease or the likelihood to die of a sickness? |
| | |
| | |
